# The knowledge and attitude of pharmacists regarding the therapeutic use of cannabinoid-based medications in Jeddah, Saudi Arabia

**DOI:** 10.1097/MD.0000000000044900

**Published:** 2025-10-31

**Authors:** Rawan H. Hareeri, Mohammed M. Aldurdunji, Abdulmohsin J. Alamoudi, Tala M. Shazli, Renad M. Ashour, Joury F. Softa, Abdulrahman A. Abusamra, Amina M. Bagher

**Affiliations:** a Department of Pharmacology and Toxicology, Faculty of Pharmacy, King Abdulaziz University, Jeddah, Saudi Arabia; b Faculty of Pharmacy, King Abdulaziz University, Jeddah, Saudi Arabia; c Department of Clinical Pharmacy, College of Pharmacy, Umm Al-Qura University, Makkah, Saudi Arabia.

**Keywords:** cannabinoids, emerging therapies, national guidelines, pharmacy education, Saudi Arabia

## Abstract

The global use of cannabinoid-based medications is expanding due to increasing evidence supporting their therapeutic potential. However, in Saudi Arabia, their adoption is influenced by cultural and regulatory factors. Pharmacists play a pivotal role in guiding the safe use of these therapies. This study aimed to assess the knowledge and attitudes of hospital pharmacists in Jeddah regarding Food and Drug Administration (FDA)-approved cannabinoid medications, identify knowledge gaps, and recommend educational strategies. A descriptive, cross-sectional, web-based survey was conducted among pharmacists from 6 randomly selected government hospitals in Jeddah, recruited through convenience-based sampling. A validated questionnaire captured demographics, knowledge of pharmacology, indications, adverse effects, interactions, and contraindications, as well as attitudes using a 5-point Likert scale. Descriptive statistics, *t*-tests, and one-way analysis of variance were applied. Of 143 respondents (49.7% male, 50.3% female), most were younger than 35 years and had 6 to 15 years of experience. Attitudes were generally moderate, with 62.3% rating the usefulness of cannabinoid therapies as moderate or high. Knowledge of adverse effects (73%), drug interactions (92%), and contraindications (88%) was stronger than recognition of FDA-approved indications (42%). A gender-related difference was noted in perceived adequacy of undergraduate preparation, with females reporting lower satisfaction (*P* = .033), though this should be considered exploratory. No other demographic factors showed significant associations. Pharmacists demonstrated moderate support for cannabinoid-based therapies and strong awareness of risks but limited recognition of approved uses. Integrated curricula, continuing professional development, and national guidelines are needed to address these gaps. Findings should be interpreted with caution, as the study was limited to public-sector hospital pharmacists.

## 1. Introduction

The discovery of endogenous cannabinoids such as anandamide and 2-arachidonoylglycerol in the early 1990s stimulated interest in *Cannabis sativa*-derived compounds for therapeutic use.^[1]^ The endocannabinoid system, which includes cannabinoid receptor type 1 and cannabinoid receptor type 2, has since been recognized as a regulator of neural signaling, immune responses, and metabolic balance.^[2]^ Preclinical studies have shown that cannabinoid receptor type 1 receptor activation modulates neurotransmitter release, while cannabinoid receptor type 2 receptor activation contributes to inflammatory control.^[3]^ Additional evidence suggests roles in neuroprotection, emotional regulation, and gastrointestinal function,^[4]^ supporting investigation of cannabinoid-based therapies in clinical practice.

Several cannabinoid-derived medicines have been approved internationally. Dronabinol and nabilone are licensed for chemotherapy-induced nausea and vomiting, while purified cannabidiol is indicated for treatment-resistant pediatric epilepsy.^[5]^ Clinical trials and systematic reviews suggest modest benefit in conditions such as multiple sclerosis-related spasticity and chronic neuropathic pain, while also raising concerns regarding dosing, tolerability, and long-term safety.^[6]^ As a result, careful patient selection and structured monitoring have been recommended for maintaining favorable benefit–risk profiles.^[6]^

In Saudi Arabia, cannabis remains prohibited under national law with no provision for medical use.^[7]^ Although the World Health Organization has encouraged balanced national policies on controlled medicines,^[8]^ no guidance has been issued by Saudi regulatory bodies or professional pharmacy councils.^[9]^ At the same time, national epidemiological data on conditions potentially relevant to cannabinoid pharmacotherapy are limited. Available reports describe chronic pain, refractory epilepsy, and cancer-related complications in tertiary care settings, but comprehensive prevalence data remain lacking.^[10,11]^ This absence of baseline information complicates assessment of clinical demand and policy development.

Pharmacy education in Saudi Arabia has not historically included structured instruction on cannabinoid pharmacotherapy. Curricular reviews have identified no dedicated modules on endocannabinoid biology, counseling, or safety.^[12]^ More broadly, training in emerging areas such as pharmacogenomics and pharmacoeconomics remains underrepresented, leaving graduates underprepared for novel clinical roles.^[13–15]^ Continuing professional development opportunities have also been reported to be insufficiently aligned with evolving pharmaceutical science.^[16]^ Regional studies indicate that pharmacists recognize the potential therapeutic value of cannabinoids but report inadequate knowledge and limited preparedness to counsel patients.^[17]^

Given the absence of formal training in cannabinoid pharmacotherapy, combined with limited epidemiological data and an uncertain regulatory framework, little is known about the readiness of pharmacists in Saudi Arabia to engage with cannabinoid-based medications if these therapies were to be introduced. Hospital pharmacists would play a central role in ensuring safe dispensing, patient counseling, and pharmacovigilance. However, no study to date has specifically examined their knowledge and attitudes in this context. The aim of this study was therefore to evaluate the knowledge and attitudes of hospital pharmacists in Jeddah toward cannabinoid-based pharmacotherapy. In doing so, the study assessed pharmacists’ knowledge of Food and Drug Administration (FDA)-approved cannabinoid medications, explored their perceptions of medical usefulness and risk–benefit balance, identified gaps in educational preparation and continuing training, and examined whether demographic or professional characteristics influenced these outcomes.

## 2. Methodology

### 2.1. Study design and setting

A descriptive, cross-sectional survey assessed knowledge and attitudes regarding cannabinoid-based therapies among hospital pharmacists in Jeddah, Saudi Arabia. Six government hospitals were selected by simple random sampling from the list of institutions under the Ministry of Health in Jeddah. These included King Abdullah Medical Complex, King Abdulaziz Hospital Al Mahjar, East Jeddah General Hospital, King Fahad General Hospital, Maternity & Children’s Hospital, and King Abdulaziz University Hospital. Data was collected between September 2023 and May 2024 to capture responses within a defined period.

### 2.2. Ethical approval and consent procedures

Approval for the study was obtained from the Research Ethical Committee of the Faculty of Medicine at King Abdulaziz University (Reference No. 411-23). Written informed consent was secured from each participant before questionnaire administration. Confidentiality was assured by anonymizing responses and storing data on a secure, access-controlled server.

### 2.3. Sample size determination and participant recruitment

The total number of pharmacists employed in government hospitals in Jeddah was used as the sampling frame. Hence, the required sample size was calculated as 143 using the Raosoft sample size calculator, applying a 95% confidence level, a 5% margin of error, and an assumed response distribution of 50%. Within each participating site, eligible pharmacists were approached during routine work hours through the department distribution lists and staff forums, ensuring coverage across inpatient, outpatient, and clinical services. Participation was voluntary, and partially completed or duplicate submissions were excluded from analysis.

### 2.4. Questionnaire development and validation

A structured questionnaire was adapted from a previously validated instrument^[17]^ and refined through content review by subject-matter experts to ensure relevance and clarity. Pilot testing (n = 15) was conducted to evaluate item comprehensibility and response burden, with minor wording adjustments implemented accordingly. Internal consistency for attitude items was acceptable, as indicated by Cronbach alpha. The final questionnaire comprised 3 sections: Demographic and professional characteristics: Age group, gender, years of professional experience, clinical role (e.g., clinical, inpatient, outpatient), hospital affiliation, and postgraduate qualifications. Attitudinal assessment: Six items addressing perceived usefulness of cannabinoid therapies, adequacy of prior education on cannabinoids, retention of knowledge, confidence in counseling about adverse effects, interactions, and contraindications. Responses were recorded on a 5-point Likert scale ranging from “very low” to “very high.” Knowledge assessment: Six domains evaluating understanding of cannabinoid pharmacology (central effects), recognition of approved cannabinoid-derived medications, identification of approved indications, common adverse effects, moderate/major drug interactions, and contraindications. Multiple-choice questions with single or multiple correct options were included; distractors were derived from literature on cannabinoid safety profiles.

### 2.5. Data collection procedures

The questionnaire was administered in a web-based format using Google Forms. Reminder notices were issued after one week and 2 weeks to improve response rate. Completion was monitored anonymously; incomplete submissions (missing > 20% of items) were excluded. Data were exported to a spreadsheet and checked for completeness and consistency.

### 2.6. Handling of missing data

Missing responses for demographic items were reported but retained if attitude and knowledge sections were complete. Participants with incomplete attitude or knowledge sections (i.e., missing responses in ≥1 item within a section) were excluded from analyses of that section. The extent and pattern of missing data were assessed; no imputation was performed, given the descriptive nature of the survey and limited missingness observed.

### 2.7. Statistical analysis

Data analysis was conducted using Minitab version 21.1 (USA). Descriptive statistics were generated for all variables, with frequencies and percentages for categorical variables and means with standard deviations for continuous or ordinal-scaled data. For each attitude item, numeric values 1 to 5 were assigned to Likert responses and item means were calculated. Knowledge items were scored as the number of correct identifications, and the percentage correct per domain was computed.

Comparative analyses evaluated associations between demographic or professional factors and attitude scores. For two-group factors (e.g., gender and postgraduate qualification), independent-samples *t* tests were used after confirming normality of item scores with the Shapiro–Wilk test. For factors with more than 2 groups (e.g., age categories, years of experience, clinical role, and hospital affiliation), one-way analysis of variance (ANOVA) was applied, with Tukey post hoc tests when the omnibus ANOVA was significant. Means (standard deviations) are reported with 95% confidence intervals (CIs). Statistical significance was set at α = 0.05.

## 3. Results

### 3.1. Participants’ demographic details

The study included the responses of 143 pharmacists in Jeddah, Saudi Arabia, from both genders: males (n = 71, 49.7%) and females (n = 72, 50.3%). The participants’ ages varied from <35 to 64 years. Pharmacists <35 years represented 52.4% (n = 75), 35 to 44 years represented 36.4% (n = 52), 45 to 54 years represented 8.4% (n = 12), and 55 to 64 years of age constituted 2.8% (n = 4). The pharmacists were grouped according to their years of professional experience. Pharmacists with less than 2 years of experience accounted for 10.5% (n = 15) of the total. Those with 2 to 5 years of experience comprised 28.7% (n = 41). Pharmacists with 6 to 15 years of experience constituted the majority at 53.8% (n = 77). Those with 16 to 25 years of experience represented 7% (n = 10), while pharmacists with over 25 years of experience comprised 0% (n = 0) of the surveyed group. The pharmacists were grouped into 4 categories based on their clinical backgrounds. The first group consisted of clinical pharmacists, comprising 16.8% (n = 24). The second largest group comprised inpatient pharmacists, making up 36.4% (n = 52). The third group consisted of outpatient pharmacists, representing 35.7% (n = 51). The final category comprised other pharmacists, accounting for 11.2% (n = 16). The participants were associated with several hospitals, with the most significant representation coming from King Abdulaziz Hospital AlMahjar 23.1% (n = 33), followed by King Abdulaziz University Hospital 22.4% (n = 32) and East Jeddah Hospital 16.8% (n = 24). Other hospitals included King Abdullah Medical Complex 16.8% (n = 24), King Fahad General Hospital Region 16.1% (n = 23), and Maternity & Children’s Hospital 4.9% (n = 7). Most participants did not have a postgraduate degree, 72.7% (n = 104), while the participants with a postgraduate degree represented only 27.3% (n = 39) (Table 1).

**Table 1:** Demographic details of the participants (N = 143).

Variable	Number of participants (%)
Gender	
Female	72 (50.3%)
Male	71 (49.7%)
Age (in yr)	
<35	75 (52.4%)
35–44	52 (36.4%)
45–54	12 (8.4%)
55–64	4 (2.8%)
Professional experience (in yr)	
<2	15 (10.5%)
2–5	41 (28.7%)
6–15	77 (53.8%)
16–25	10 (7.0%)
+25	0 (0%)
Clinical background	
Clinical pharmacist	24 (16.8%)
Inpatient pharmacist	52 (36.4%)
Outpatient pharmacist	51 (35.7%)
Other	16 (11.2%)
Name of hospital	
East Jeddah Hospital	24 (16.8%)
King Abdulaziz Hospital Al Mahjar	33 (23.1%)
King Abdulaziz University Hospital	32 (22.4%)
King Abdullah Medical Complex	24 (16.8%)
King Fahad General Hospital region	23 (16.1%)
Maternity & Children’s Hospital	7 (4.9%)
Postgraduate degree	
Yes	39 (27.3%)
No	104 (72.7%)

### 3.2. Participants’ attitudes towards medicinal cannabinoids

The attitudes of participants regarding the usefulness and educational aspects of medicinal cannabinoids were mixed. Most respondents agreed that cannabinoids have medical usefulness, and attitudes toward the usefulness of FDA-approved cannabis-derived drugs were generally positive. However, lower levels of agreement were reported for items assessing educational exposure, retention of knowledge from undergraduate training, and confidence in educating patients regarding adverse effects, contraindications, and drug interactions. For example, when asked whether they had learned enough about medicinal cannabinoids during undergraduate studies, 67 participants (46.9%) selected moderate agreement, 34 (23.8%) selected high or very high agreement, and 42 (29.4%) selected low or very low agreement. Overall, medicinal cannabinoids were generally regarded as medically useful.

Lower levels of agreement were observed for items addressing educational sufficiency and knowledge retention. For instance, only 31 participants (21.7%) reported high or very high agreement that they adequately remember what they learned, whereas 42 (29.4%) indicated low or very low agreement. Preparedness to counsel patients on adverse effects, contraindications, and drug–drug interactions also showed a similar distribution, with 42 participants (29.4%) selecting high or very high agreement, compared with 70 (49.0%) who selected low or very low agreement. Overall, while respondents acknowledged the potential medical usefulness of cannabinoids, deficiencies were reported in educational exposure, retention of knowledge, and preparedness for clinical application, as presented in Table 2.

**Table 2:** Attitudes of participants regarding the useful and educational aspects of medicinal cannabinoids (N = 143).

Questions	Level of participant agreement number (%)				
	Very low	Low	Moderate	High	Very high
Do you think that cannabis has medical usefulness?	9 (6.3%)	32 (22.4%)	54 (37.8%)	35 (24.5%)	13 (9.1%)
Do you think cannabis-derived FDA-approved drugs are useful?	9 (6.3%)	25 (17.5%)	51 (35.7%)	44 (30.8%)	14 (9.8%)
Do you think medicinal cannabinoid benefits outweigh their risks?	12 (8.4%)	30 (21.0%)	67 (46.9%)	25 (17.5%)	9 (6.3%)
Do you think you learned enough about medicinal cannabinoids during your undergraduate studies?	37 (25.9%)	41 (28.7%)	52 (36.4%)	8 (5.6%)	5 (3.5%)
Do you think you adequately remember what you learned?	27 (18.9%)	43 (30.1%)	50 (35.0%)	19 (13.3%)	4 (2.8%)
Do you think you are knowledgeable enough to educate patients on the adverse effects, interactions, and contraindications of cannabinoids?	17 (11.9%)	38 (26.6%)	53 (37.1%)	27 (18.9%)	8 (5.6%)

### 3.3. Factors influencing participants’ attitudes regarding medicinal cannabinoids

Table 3 illustrates the influence of demographic factors on participants’ attitudes toward medicinal cannabinoids. The data presented as a subgroup means with 95% CIs to convey precision alongside *P*-values. A significant association was observed between gender and the perception of receiving adequate education about medicinal cannabinoids during undergraduate studies, with females reporting lower satisfaction (mean 2.63, 95% CI: 2.32–2.94) compared to males (mean 3.07, 95% CI: 2.79–3.35; *P* = .033) (Table 3). However, gender differences were not significant for other parameters, such as the belief in cannabis’s medical usefulness (*P* = .135), the usefulness of FDA-approved cannabis-derived drugs (*P* = .389), or knowledge adequacy in educating patients (*P* = .12).

**Table 3:** Influence of demographic factors on the attitudes of participants (N = 143).

Characteristics	No.	Do you think that cannabis has medical usefulness?			Do you think cannabis-derived FDA-approved drugs are useful?			Do you think medicinal cannabinoid benefits outweigh their risks?			Do you think you learned enough about medicinal cannabinoids during your undergraduate studies?			Do you think you adequately remember what you learned?			Do you think you are knowledgeable enough to educate patients on the adverse effects, interactions, and contraindications of cannabinoids?		
		Mean (SD)	(95% CI)	*P*-value	Mean (SD)	(95% CI)	*P*-value	Mean (SD)	(95% CI)	*P*-value	Mean (SD)	(95% CI)	*P*-value	Mean (SD)	(95% CI)	*P*-value	Mean (SD)	(95% CI)	*P*-value
Female	72	3.64 (1.1)	3.38–3.90	.135	3.56 (1.13)	3.29–3.83	.389	3.28 (1.1)	3.02–3.54	.579	2.63 (1.3)	2.32–2.94	.033*	3.03 (1.3)	2.72–3.34	.206	3.19 (1.2)	2.91–3.47	.12
Male	71	3.35 (1.2)	3.07–3.63		3.48 (1.27)	3.18–3.78		3.38 (1.3)	3.07–3.69		3.07 (1.2)	2.79–3.35		3.3 (1.2)	3.02–3.58		3.51 (1.2)	3.23–3.79	
<35	75	3.533 (1.09)	3.28–3.78	.235	3.67 (1.1)	3.42–3.92	.395	3.25 (1.1)	3.00–3.50	.395	2.91 (1.2)	2.63–3.19	.347	3.00 (1.2)	2.72–3.28	.278	3.29 (1.3)	2.99–3.59	.528
35–44	52	3.308 (1.25)	2.96–3.66		3.23 (1.3)	2.87–3.59		3.31 (1.1)	3.00–3.62		2.87 (1.3)	2.51–3.23		3.33 (1.3)	2.97–3.69		3.31 (1.1)	3.00–3.62	
45–54	12	3.833 (0.98)	3.21–4.46		4.00 (1.0)	3.36–4.64		3.83 (0.9)	3.26–4.40		2.75 (1.4)	1.86–3.64		3.58 (1.2)	2.82–4.34		3.67 (1.2)	2.91–4.43	
55–64	4	4.25 (0.96)	2.72–5.78		4.75 (0.5)	3.95–5.55		3.50 (1.9)	0.48–6.52		1.75 (1.5)	−0.64–4.14		2.75 (2.1)	−0.59–6.09		4.00 (1.2)	2.09–5.91	
<2	15	3.40 (1.2)	2.74–4.06	.652	4.00 (1.1)	3.39–4.61	.352	3.20 (1.3)	2.48–3.92	.403	2.67 (1.5)	1.84–3.50	.388	2.47 (1.1)	1.86–3.08	.166	3.13 (1.2)	2.47–3.79	.878
2–5	41	3.66 (0.9)	3.38–3.94		3.49 (1.1)	3.14–3.84		3.49 (0.8)	3.24–3.74		3.02 (1.1)	2.67–3.37		3.22 (1.1)	2.87–3.57		3.39 (1.1)	3.04–3.74	
6–15	77	3.47 (1.3)	3.17–3.77		3.48 (1.3)	3.18–3.78		3.22 (1.2)	2.95–3.49		2.86 (1.2)	2.59–3.13		3.25 (1.3)	2.95–3.55		3.35 (1.2)	3.08–3.62	
16–25	10	3.20 (1.0)	2.48–3.92		3.90 (1.2)	3.04–4.76		3.70 (1.1)	2.91–4.49		2.30 (1.6)	1.16–3.44		3.30 (1.4)	2.30–4.30		3.50 (1.4)	2.50–4.50	
Clinical pharmacist	24	3.75 (1.4)	3.16–4.34	.294	3.63 (1.2)	3.12–4.14	.991	3.21 (1.2)	2.70–3.72	.069	3.13 (1.4)	2.54–3.72	.365	3.29 (1.5)	2.66–3.92	.821	3.25 (1.2)	2.74–3.76	.15
Inpatient pharmacist	52	3.33 (1.0)	3.05–3.61		3.56 (1.2)	3.23–3.89		3.17 (1.0)	2.89–3.45		2.64 (1.2)	2.31–2.97		3.04 (1.2)	2.71–3.37		3.10 (1.3)	2.74–3.46	
Outpatient pharmacist	51	3.63 (1.1)	3.32–3.94		3.57 (1.3)	3.20–3.94		3.65 (1.1)	3.34–3.96		2.96 (1.2)	2.62–3.30		3.24 (1.2)	2.90–3.58		3.63 (1.1)	3.32–3.94	
Other	16	3.25 (1.4)	2.50–4.00		3.50 (1.3)	2.81–4.19		3.00 (1.0)	2.47–3.53		2.75 (1.4)	2.00–3.50		3.13 (1.2)	2.49–3.77		3.44 (1.2)	2.80–4.08	
King AbdulAziz Hospital Al Mahjar	33	3.55 (1.2)	3.12–3.98	.086	3.58 (1.2)	3.15–4.01	0.889	3.46 (1.0)	3.11–3.81	.163	2.91 (1.3)	2.45–3.37	.486	3.61 (1.1)	3.22–4.00	.118	3.67 (1.1)	3.28–4.06	.058
East Jeddah Hospital	24	3.54 (1.1)	3.08–4.00		3.46 (1.3)	2.91–4.01		3.29 (0.9)	2.91–3.67		2.46 (1.3)	1.91–3.01		2.88 (1.4)	2.29–3.47		3.08 (1.4)	2.49–3.67	
King Abdullah Medical Complex	24	2.88 (1.2)	2.37–3.39		3.38 (1.4)	2.79–3.97		2.79 (1.4)	2.20–3.38		3.13 (1.2)	2.62–3.64		2.83 (1.2)	2.32–3.34		3.17 (1.3)	2.62–3.72	
King AbdulAziz University Hospital	32	3.69 (1.1)	3.29–4.09		3.72 (1.2)	3.29–4.15		3.56 (1.0)	3.20–3.92		2.91 (1.4)	2.41–3.41		3.03 (1.4)	2.53–3.53		3.34 (1.2)	2.91–3.77	
King Fahad General Hospital region	23	3.78 (0.9)	3.39–4.17		3.57 (1.0)	3.14–4.00		3.44 (1.0)	3.01–3.87		2.91 (1.1)	2.43–3.39		3.44 (1.2)	2.92–3.96		3.70 (0.8)	3.35–4.05	
Maternity & Children’s Hospital	7	3.43 (1.0)	2.51–4.35		3.86 (1.2)	2.75–4.97		3.29 (1.4)	2.00–4.58		2.43 (1.0)	1.51–3.35		2.86 (0.9)	2.03–3.69		2.29 (1.0)	1.37–3.21	
Yes	39	3.49 (1.5)	3.00–3.98	0.96	3.62 (1.4)	3.17–4.07	0.783	3.33 (1.1)	2.97–3.69	.976	2.87 (1.5)	2.38–3.36	.893	3.33 (1.3)	2.91–3.75	.321	3.23 (1.2)	2.84–3.62	.477
No	104	3.50 (1.0)	3.31–3.69		3.55 (1.2)	3.32–3.78		3.33 (1.1)	3.12–3.54		2.84 (1.2)	2.61–3.07		3.10 (1.3)	2.85–3.35		3.39 (1.2)	3.16–3.62	

Means are presented with 95% confidence intervals. Group comparisons used *t*-tests for two-level factors and one-way ANOVA with Tukey post hoc tests; statistical significance was set at *P* < .05.

Age was not significantly associated with attitudes across all parameters, with *P*-values ranging from .235 to .528. Similarly, professional experience did not significantly impact participants’ perceptions, though participants with 2 to 5 years of experience reported slightly higher mean scores across most categories (*P*-values: .166–.878).

No significant differences were observed based on clinical background, including the perception of whether the benefits of medicinal cannabinoids outweigh the risks (*P* = .069). Hospital affiliation did not show a strong association with participants’ attitudes. However, King Fahad General Hospital participants reported higher mean scores for knowledge adequacy (3.70), but this did not reach statistical significance (*P* = .058).

Lastly, holding a postgraduate degree was not significantly associated with participants’ attitudes, with *P*-values ranging from .321 to .976. These results indicate that gender had the most notable influence on participants’ perceptions, specifically regarding their undergraduate education on medicinal cannabinoids. At the same time, other demographic factors had limited or no significant associations.

### 3.4. Participants’ knowledge of medicinal cannabinoids

After collecting the demographic details and attitudes towards medicinal cannabinoids, participants were asked questions about their knowledge of medicinal cannabinoids. They were first asked to identify the central pharmacological effects of cannabinoids, with options including “Depressant,” “Stimulant,” and “Hallucinogenic.” The majority answered hallucinogenic, 45.7% (n = 84), while 32.6% (n = 60) selected depressant, and 21.7% (n = 40) answered stimulant. Participants were then asked to choose FDA-approved cannabinoid-based drugs based on their knowledge and were given the following options: “Cesamet®” which consisted of 28.9% (n = 61), followed by “Epidiolex®,” 11.4% (n = 24), then “Marinol®” represented 34.6% (n = 73), and finally “Syndros®” 25.1% (n = 53). The participants knowledge were then assessed in the following categories: FDA-approved indications, adverse effects, moderate or major interactions, and contraindications. They were given options with correct and incorrect answers and were asked to identify the correct ones. Options were provided, and each participant was allowed to choose one, multiple, or all answers. Correct identification rates for cannabis/cannabinoid FDA-approved drug indications, common adverse effects, drug interactions, and cautions/contraindications were 42%, 73%, 92%, and 88%, respectively. Conversely, the incorrect identification rates for these categories were 58%, 27%, 8%, and 12%, respectively. Overall, 74% of participants demonstrated accurate knowledge of cannabinoid FDA-approved drug indications, adverse effects, interacting drugs, and contraindications. In comparison, an overall error rate of 26% was observed due to incorrect responses in these areas. A full breakdown of these knowledge items and participants’ responses is presented in Table 4.

**Table 4:** Participants’ knowledge of medical cannabinoids.

Questions and answers	Number (%)
What central pharmacological effects do cannabis or cannabinoids have?	
Depressant	60 (32.6%)
Hallucinogenic	84 (45.7%)
Stimulant	40 (21.7%)
Total number	184
Which of the following FDA-approved cannabinoid-based drugs do you know?	
Cesamet®	61 (28.9%)
Epidiolex®	24 (11.4%)
Marinol®	73 (34.6%)
Syndros®	53 (25.1%)
Total number	211
Which indications are cannabinoid-based drugs approved by the FDA?	
Anorexia in AIDS patients*	33 (9.6%)
Chronic neuropathic pain†	74 (21.4%)
Nausea associated with cancer chemotherapy*	52 (15.1%)
Pain associated with cancer†	68 (19.7%)
Resistant major depressive disorder†	28 (8.1%)
Seizures in Lennox–Gastaut syndrome*	59 (17.1%)
Weight loss in obese patients†	31 (9.0%)
Total number	345
Which adverse effects/events are commonly caused by cannabis/cannabinoids?	
Anemia†	11 (2.6%)
Anxiety*	62 (14.8%)
Constipation†	25 (6.0%)
Glaucoma†	17 (4.1%)
Hyperglycemia†	14 (3.3%)
Insomnia*	42 (10.0%)
Loss of appetite†	46 (11.0%)
Memory/cognitive impairment*	79 (18.9%)
Orthostatic hypotension*	51 (12.2%)
Tachycardia*	71 (17.0%)
Total number	418
Which drugs have moderate or major interactions with cannabis/cannabinoids?	
Acetaminophen†	7 (1.8%)
Alprazolam*	47 (12.1%)
Amoxicillin†	11 (2.8%)
Diphenhydramine*	27 (7.0%)
Fluoxetine*	69 (17.8%)
Ibuprofen†	6 (1.5%)
Omeprazole†	9 (2.3%)
Phenytoin*	63 (16.2%)
Pregabalin*	78 (20.1%)
Warfarin*	71 (18.3%)
Total number	388
Which conditions are cautions/contraindications for cannabis/cannabinoid usage?	
Cancer†	15 (4.2%)
Cardiac arrhythmia*	72 (20.3%)
Cardiovascular disorders*	71 (20.1%)
Glaucoma†	13 (3.7%)
Hypothyroidism†	16 (4.5%)
Major depressive disorder*	41 (11.6%)
Pregnancy*	84 (23.7%)
Schizophrenia*	42 (11.9%)
Total number	354

* Correct.

† Incorrect.

## 4. Discussion

In this study, 89 of 143 participants (62.3%) reported moderate or high perceptions of cannabis usefulness, whereas only 60 of 143 (42.0%) correctly identified FDA-approved therapeutic indications. By contrast, risk awareness was stronger, with 104 of 143 (73.0%) identifying adverse effects and 132 of 143 (92.3%) identifying drug interactions. Preparedness to counsel patients was limited, as 55 of 143 (38.5%) reported low or very low confidence. Female pharmacists also reported lower confidence in the adequacy of their undergraduate training, suggesting a possible gender-related difference in perceived preparedness.

The imbalance between recognition of usefulness and stronger awareness of risks appears consistent with reports from other prohibition contexts. In Jordan, pharmacists expressed interest in the therapeutic potential of cannabis but also reported substantial knowledge gaps and limited confidence in counseling, with attitudes shaped by concerns over safety and misuse.^[17–19]^ Systematic reviews of health professionals similarly suggest that uncertainty regarding efficacy and safety often outweighs comfort with therapeutic use.^[20]^ These findings are broadly comparable to the present study, where most participants recognized risks but fewer than half identified therapeutic indications, pointing to a knowledge imbalance that may be reinforced by prohibition and the absence of curricular training.

In partial legalization contexts such as the United States, pharmacists seem to demonstrate greater recognition of therapeutic benefits but continue to report deficits in confidence and clarity regarding legal frameworks. Surveys have noted that while a large proportion acknowledged the usefulness of medical cannabis, many did not feel sufficiently knowledgeable to counsel patients.^[21]^ Confusion about legislation and reliance on informal learning sources were also reported, patterns that may echo the uncertainty observed in this study.^[22,23]^ This suggests that exposure to legalized cannabis can increase awareness of potential benefits but does not necessarily overcome barriers to patient counseling.

In legalized contexts such as Canada and Australia, pharmacists generally report higher recognition of therapeutic indications, yet significant gaps in counseling preparedness remain. In Canada, approximately two-thirds of pharmacists indicated the need for further education despite legalization.^[22]^ Qualitative research likewise suggested that pharmacists were more comfortable dispensing cannabis products but still perceived gaps in training related to safety and efficacy.^[23]^ In Australia, pharmacists identified inadequate understanding of legislative requirements and limited preparation for patient counseling.^[18]^ Taken together, these studies imply that legalization may enhance familiarity with therapeutic roles but does not, in itself, ensure clinical preparedness.

Confidence in patient counseling also appears limited among pharmacy students, with surveys in North America reporting inadequate curricular exposure and a strong demand for expanded training.^[24,25]^ Proposals for experiential and case-based learning may improve preparedness,^[24]^ while professional associations have noted that pharmacists remain underutilized in patient education due to weaknesses in training frameworks.^[26]^ These observations are consistent with the present findings, where risk recognition was high but preparedness to counsel patients was low.

The gender-related difference observed in this study should be interpreted cautiously. Although female pharmacists reported lower confidence in the adequacy of their training, pharmacy education in Saudi Arabia provides equivalent access across genders. Literature in health professions education suggests that male students often rate their competence more highly, whereas female students are more likely to underestimate their abilities.^[27,28]^ Self-assessment accuracy also may not improve with seniority.^[29]^ These patterns have been described as “confidence misalignment,” where perceptions of competence diverge from actual ability.^[30]^ While such explanations are plausible, further research would be needed to clarify whether the observed differences in this study reflect self-assessment bias, sociocultural influences, or unaddressed educational needs.

The educational implications of these findings are noteworthy. The limited recognition of approved indications and low counseling preparedness may indicate a need for structured reform in pharmacy curricula and professional development. Similar gaps have been documented in Saudi pharmacy education in relation to pharmacogenomics, pharmacoeconomics, and public health.^[13–15]^ Studies of targeted training programs in areas such as anemia and diabetes management suggest that structured education can improve pharmacist preparedness.^[31,32]^ Moreover, research during the COVID-19 pandemic showed that pharmacists in Saudi Arabia and Jordan were willing to assume frontline roles but reported inadequate training and low confidence in public health responsibilities.^[16,33,34]^ These parallels imply that the gaps observed in this study may not be unique to cannabinoid pharmacotherapy but instead reflect broader systemic challenges in pharmacy education and continuing professional development.

This study has limitations. It was restricted to hospital pharmacists in public-sector institutions within a single metropolitan region, which may limit the generalizability of findings to other practice settings. The cross-sectional design precludes causal inference, and reliance on self-reported knowledge and attitudes introduces the potential for reporting bias and may not fully reflect actual competence. Nonetheless, the study provides important baseline evidence on pharmacists’ preparedness for cannabinoid pharmacotherapy in Saudi Arabia.

Future research could include pharmacists from diverse practice settings and make use of longitudinal and qualitative approaches to evaluate educational interventions. Further exploration of gender-related differences in self-assessment may also be informative. Such work may contribute to curriculum development and professional training, supporting pharmacists’ preparedness for cannabinoid pharmacotherapy should regulatory changes occur. These findings may also be relevant to ongoing healthcare reforms in Saudi Arabia, where national authorities are considering frameworks for emerging therapies.

## 5. Conclusion

The findings of this study suggest that hospital pharmacists in Jeddah recognize the potential medical usefulness of cannabinoids but demonstrate stronger awareness of risks than of approved therapeutic indications, alongside limited confidence in patient counseling. These patterns align with international evidence, where knowledge imbalances and educational gaps are common regardless of legal status. The results indicate a systemic need to strengthen pharmacy curricula, continuing professional development, and the establishment of national guidelines in Saudi Arabia to ensure preparedness should cannabinoid pharmacotherapy become clinically relevant. Further research across diverse settings and using mixed-method approaches would be valuable to clarify determinants of confidence, educational adequacy, and gender-related differences in self-assessment, while expanding the evidence base to private and community practice environments.

## Acknowledgments

The authors wish to thank all who gave their time and participated in this study. The authors, therefore, acknowledge with thanks WAQF and the Deanship of Scientific Research (DSR) for technical and financial support.

## Author contributions

**Conceptualization:** Rawan H. Hareeri, Abdulmohsin J. Alamoudi, Tala M. Shazli, Renad M. Ashour, Joury F. Softa, Abdulrahman A. Abusamra, Amina M. Bagher.

**Data curation:** Joury F. Softa, Abdulrahman A. Abusamra.

**Formal analysis:** Rawan H. Hareeri, Mohammed M. Aldurdunji, Abdulmohsin J. Alamoudi, Tala M. Shazli, Renad M. Ashour, Abdulrahman A. Abusamra.

**Investigation:** Rawan H. Hareeri, Tala M. Shazli, Renad M. Ashour, Joury F. Softa.

**Methodology:** Rawan H. Hareeri, Mohammed M. Aldurdunji, Abdulmohsin J. Alamoudi, Renad M. Ashour, Joury F. Softa, Abdulrahman A. Abusamra, Amina M. Bagher.

**Project administration:** Rawan H. Hareeri, Mohammed M. Aldurdunji, Abdulmohsin J. Alamoudi, Tala M. Shazli, Amina M. Bagher.

**Resources:** Tala M. Shazli, Joury F. Softa, Abdulrahman A. Abusamra.

**Software:** Mohammed M. Aldurdunji, Renad M. Ashour, Joury F. Softa.

**Supervision:** Rawan H. Hareeri, Abdulmohsin J. Alamoudi, Amina M. Bagher.

**Validation:** Rawan H. Hareeri, Abdulmohsin J. Alamoudi, Abdulrahman A. Abusamra.

**Writing – original draft:** Rawan H. Hareeri, Mohammed M. Aldurdunji, Abdulmohsin J. Alamoudi, Tala M. Shazli, Renad M. Ashour, Joury F. Softa, Abdulrahman A. Abusamra, Amina M. Bagher.

**Writing – review & editing:** Rawan H. Hareeri, Mohammed M. Aldurdunji, Abdulmohsin J. Alamoudi, Tala M. Shazli, Renad M. Ashour, Joury F. Softa, Abdulrahman A. Abusamra, Amina M. Bagher.
